# Effect of chamomile intake on blood coagulation tests in healthy volunteers: a randomized, placebo-controlled, crossover trial

**DOI:** 10.1186/s13741-023-00339-7

**Published:** 2023-09-20

**Authors:** Jonathon A. Schwartz, Jamie L. Romeiser, Reona Kimura, Lisa Senzel, Dennis Galanakis, Darcy Halper, Shayla Mena, Elliott Bennett-Guerrero

**Affiliations:** 1 Department of Anesthesiology, Health Sciences Center, Level 4, Stony Brook University Hospital, Stony Brook University, 101 Nicolls Road, Stony Brook, NY 11794-8434 USA; 2 Renaissance School of Medicine, Stony Brook University, Stony Brook, NY USA; 3 Department of Pathology, Stony Brook University, Stony Brook, NY USA

**Keywords:** Chamomile, Coagulation, Prothrombin time, Herbal, And Tea

## Abstract

**Background:**

Chamomile is consumed worldwide for enjoyment and its potentially desirable properties. Widespread patient resource websites, however, discourage preoperative chamomile intake, lest bleeding could worsen. This precaution, though, stems largely from indirect evidence in one case report. To evaluate if chamomile ingestion impacts coagulation assays via coumarin-like substances, we designed a randomized, placebo-controlled, crossover study.

**Materials and methods:**

Healthy volunteers were randomized to three interventions in a cross-over-design spanning 5 weeks per subject. Interventions included 7-day consumption of chamomile tea (3 tea bags × 3 times daily = 9 tea bags daily), a chamomile extract capsule (3 times daily), or a placebo capsule (3 times daily). A 7-day washout period elapsed between intervention periods. The primary outcome was the change in prothrombin time (PT) before vs. after each intervention. Secondary outcomes included changes in the international normalized ratio (INR), activated partial thromboplastin time (aPTT), thrombin time (TT), reptilase time (RT), and fibrinogen (FG) surrounding each intervention.

**Results:**

All 12 enrolled subjects were randomized and completed the study. The primary outcome of PT change (mean ± SD) was similar across interventions (chamomile tea =  − 0.2 ± 0.4 s, extract capsule =  − 0.2 ± 0.4 s, and placebo capsule = 0.1 ± 0.5 s; *p* = 0.34). INR change was 0 s (*p* = 0.07) for each intervention. The aPTT, TT, RT, and FG, did not change significantly across interventions (*p* = 0.8, *p* = 0.08, *p* = 0.8, and *p* = 0.2 respectively).

**Conclusions:**

Chamomile intake by tea or capsule does not prolong PT. These findings challenge the notion to avoid perioperative chamomile intake in patients not taking warfarin.

**Trial registration:**

ClinicalTrials.gov, NCT05006378; Principal Investigator: Jonathon Schwartz, M.D.; Registered August 16, 2021.

## Background

Chamomile is a widely consumed herb that may possess desirable properties and benefits (Srivastava et al. 2010). Two key varieties of chamomile, German Chamomile (*Matricaria chamomilla*) and Roman Chamomile (*Chamaemelum nobile*), are the most prevalent in consumer products (Sagi et al. 2014). Chamomile is usually ingested as a tea but may also be ingested as an extract capsule or as a tincture. Numerous patient information sources, however, have advised against preoperative chamomile intake out of concern for raising surgical bleeding risk. One example of this warning is from a large health system in New York; their website states “Stop taking chamomile at least 2 weeks before surgery or dental surgery, because of the risk of bleeding.” (Mount Sinai 2022). In another example, a popular health website, which has 30 million visits per month, warns patients that “Due to concerns about bleeding, chamomile shouldn't be used two weeks before or after surgery.” (Wong 2022). Of note, these precautions are broad and are *not* limited to individuals receiving anticoagulants.

**Fig. 1:**
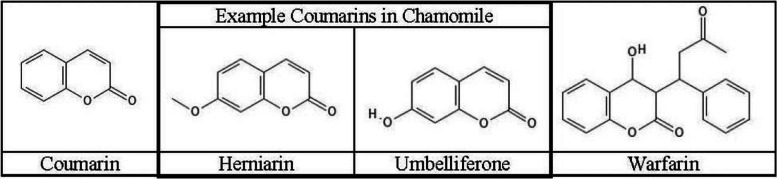
Example phytocoumarins present in chamomile and their structural relationship to warfarin. Shown are the structures for two coumarin compounds prevalent in chamomile flowers (herniarin and umbelliferone), along with the parent structural compound, coumarin. The well-known anticoagulant derivative of coumarin, warfarin (absent in chamomile), is also shown to illustrate structural analogy to the example phytocoumarins

These recommendations, however, appear to be based upon two relatively weak pieces of evidence. First, it is known that chamomile contains coumarin-like substances, such as herniarin and umbelliferone (Fig. 1) (Srivastava et al. 2010; Tschiggerl and Bucar 2012; Petruľová-Poracká et al. 2013). The presence of these phytochemicals in chamomile could provide some pharmacologic justification or mechanism by which chamomile might cause bleeding. Second, clinical evidence for the risk appears to be based largely on a single case report of spontaneous internal hemorrhage in an elderly woman receiving warfarin and consuming large amounts of chamomile tea (Segal and Pilote 2006). The patient’s international normalized ratio (INR) was very elevated (7.9) despite confirmation of appropriate warfarin dosing, a stable diet, and a history of stable INR beforehand on the same dose. Since chamomile possesses coumarin-like substances, the authors of this case report speculated that coumarins from chamomile may have contributed toward the spontaneous internal hemorrhage, possibly via synergy with warfarin or via drug-drug interactions.

Therefore, given the clinical relevance of this issue, i.e., potential bleeding, and the lack of any rigorous studies on this subject, we designed a randomized, placebo-controlled, crossover study to test the hypothesis that chamomile ingestion by volunteers not taking anticoagulants impacts coagulation assays.

## Methods

This study was approved by the local Institutional Review Board (IRB # 2021–00314) and written informed consent was obtained from all subjects participating in the trial. The trial was registered prior to patient enrollment at clinicaltrials.gov. The study was conducted between August 31, 2021, and January 19, 2022, when the last enrolled subject completed the final follow-up visit. The manuscript was prepared according to the applicable Consolidated Standards of Reporting Trials (CONSORT) guidelines (Gagnier et al. 2006).

### Study participants

Inclusion criteria were (1) adults at least 18 years of age up to 75 years of age, (2) able to provide informed written consent, and (3) willing to withhold use of chamomile products outside of the research study.

Exclusion criteria were (1) a past medical or family history of bleeding or thrombotic disorders; (2) taking chronic medications known to affect hemostasis, such as anticoagulants, antiplatelet agents, and selective-serotonin reuptake inhibitors; (3) a history of an abnormally elevated INR, prothrombin time (PT), activated partial thromboplastin time (aPTT), thrombin time (TT), or reptilase time (RT); (4) more than weekly non-steroidal anti-inflammatory drug use, e.g., aspirin, ibuprofen, or naproxen. (5) active intake of either ginger, garlic, ginkgo, ginseng, fish oil, black cohosh, feverfew, valerian, coenzyme q10, goldenseal, St. John Wort within 14 days of enrollment; (6) severe allergy to ragweed; (7) reported chamomile allergy; (8) consuming three or more alcoholic beverages daily; (9) active cigarette smoking; (10) female subjects who were pregnant, breast-feeding, or lactating; (11) hospitalization at time of screening; (12) scheduled surgical procedure during study period; (13) underweight subjects with body mass index < 18 kg/m^2^) or a history of malnourishment; (14) symptoms of active infection; (15) a history of estrogen-dependent condition such as uterine fibroids, breast cancer, uterine cancer, or ovarian cancer; (16) active intake of cyclosporine.

After informed written consent was obtained, baseline screening was completed and subjects were excluded (screen failed) if (1) the baseline complete blood count demonstrated a hematocrit below 30%, (2) platelet count below 150 × 10^3^/μL, (3) white blood cell count below 3 × 10^3^/μL or above 15 × 10^3^/μL.

### Objectives and hypothesis

Our primary objective was to test the hypothesis that ingestion of chamomile prolongs the prothrombin time.

### Study interventions

#### Summary of design

**Fig. 2:**
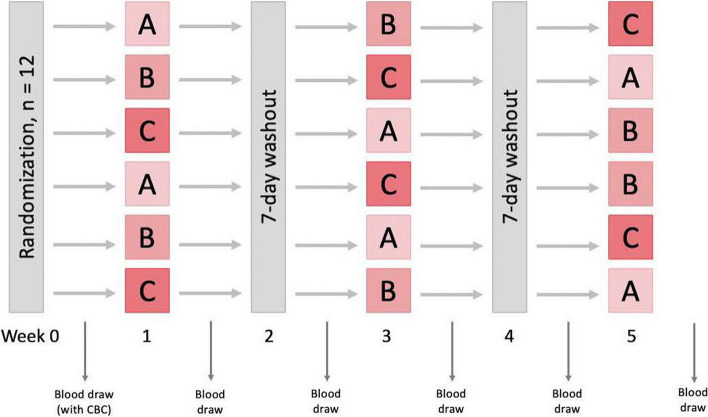
Randomized order of interventions and laboratory sampling time points. Letters “A,” “B,” and “C” represent the three interventions (A = chamomile tea, B = chamomile capsule, and C = placebo capsule), therefore, there were 6 possible orders of interventions that subjects could be randomized to. A screening sample was collected within 72 h prior to initiation of the first study intervention (week 1). Subsequent blood samples were obtained before and after completing each 7-day (i.e., weeklong) intervention

The study was a randomized, controlled cross-over design occurring over 5 weeks per subject (Fig. 2). After consent and screening tests, all subjects received the following three 7-day long interventions (3 chamomile teabags 3 times per day, 1 chamomile extract capsules 3 times per day, and 1 placebo capsules 3 times per day). Blood samples were obtained at the beginning and end of each weeklong intervention and sent to our hospital’s coagulation laboratory for analysis (see details below). There was a 1-week washout period in-between interventions. Subjects were randomized to the order in which the 3 interventions were received (see “Statistical methods” section and Fig. 2).

#### Study products and chamomile preparations

##### Chamomile tea drink

Subjects were instructed to consume a cup (approximately 5 oz.) of chamomile tea three times a day (morning, daytime, and evening). Tea was prepared using a widely available mass-produced tea bag (Celestial Seasonings®, USA). A total of three tea bags (1 g of dried flower in each tea bag) was used to make the cup of tea, which thus contained 3 g of tea. Therefore, over the course of the day subjects consumed 3 cups of tea, which contained a total tea dose of 9 g. The tea was steeped in either boiling water, water dispensed by an electric kettle that completes its heating cycle, or an on-demand heated water dispenser. Subjects were instructed that they should observe steam emerging from the cup once it is dispensed. Each dose of three tea bags (3 g total dose) was steeped in the 5 oz of hot water for 10 min. After the steeping period, tea bags were removed. Sugar and artificial sweeteners using aspartame, sucralose, or stevia extract were permitted as there is no established interaction between chamomile ingredients and sweeteners. Subjects were instructed to avoid adding other substances such as milk to the teacup during consumption. Subjects were also instructed to consume the tea shortly after meals, and to maintain their usual dietary habits. Subjects completed a daily log of the times they ingested the relevant tea and capsule (placebo or chamomile) as described below.

##### Chamomile powder extract in capsules

During a separate week (Fig. 2) subjects consumed chamomile extract capsules. The extract capsules were purchased from a single supplier, Swanson Superior Herbs- Chamomile Flower Extract-Standardized Apigenin 1.2% (Swanson Health Products, USA). Each of these capsules contains 500 mg of a chamomile extract which has been standardized by the manufacturer to 1.2% apigenin content by weight. A certificate of authenticity from a third-party analyst (Health Wright Products Inc., USA) was obtained from the capsule provider to ensure appropriate chamomile composition. To mask the content of the capsules (visually and odor), our hospital’s Investigational Pharmacy inserted the chamomile capsules into opaque blue capsules (Article No. 800223, Fagron Inc., USA). Subjects were instructed to consume one 500 mg capsule three times a day for a total daily dose of 1500 mg.

##### Placebo capsules

During a separate week (Fig. 2) subjects consumed a week of placebo capsules. Placebo capsules were prepared by our hospital’s Investigational Pharmacy using methylcellulose filler (Fagron Inc., USA) inserted into the same opaque blue container capsules used for the active drug and sealed to ensure blinding from compound appearance and smell.

#### Blood sampling

Venous blood samples were collected by trained study staff, using sterile technique and a 21-gauge phlebotomy needle, at six time points throughout the study period (Fig. 2): (1) screening within 72 h prior to initiation of the first study intervention (day 0), (2) after completing the first 7-day intervention week (morning of day 8), (3) after 7 days washout of initial exposure, (4) after 7 days of second 7-day intervention, (5) after 7 days washout from second intervention, and (6) after 7 days of the third 7-day intervention.

At each of these 6 phlebotomy visits, 15 ml of blood were collected into citrated blood tubes (BD Vacutainer® Buff. Na Citrate 0.109 M, 3.2%, Becton, Dickinson and Company, USA). The blood tubes were delivered to our hospital’s Coagulation Clinical Laboratory within 2 h of sampling, which is earlier than the 4-h timeframe required by the laboratory. The blood samples were centrifuged in the laboratory to obtain platelet-poor plasma. Coagulation assays were completed on the platelet-poor plasma using the ACL TOP 750 CTS automatic coagulation analyzer (Werfen, Bedford, MA). Complete blood counts were performed on uncentrifuged blood using the Sysmex XN-10 hematology analyzer (Sysmex America, Lincolnshire, IL, USA).

### Endpoints and statistical methods

Data for this trial were entered into a dedicated REDCap database (REDCap, USA), which includes numerous safeguards to protect the integrity of the data. For example, logs are generated for all entry, revision, and deletion of data. All statistical analyses were performed by the study’s statistician (co-author JR).

The primary endpoint for the study was the change in PT from the baseline value at the start of each randomized 7-day long intervention. Secondary endpoints for the study included changes in the INR, aPTT, TT, RT, and FG from baseline values at the start of each randomized intervention. Safety endpoints included new easy bruising, new gum bleeding, or any new bleeding event occurring during the study. A separate standardized questionnaire including questions unrelated to coagulation or bleeding, e.g., sleep quality, was also obtained, and the results will be reported separately.

### Randomization and design

Subjects were randomized into a William’s square design, which is a special cross-over study design used to balance for carryover effects (Wang et al. 2009). Three 7-day long interventions (Fig. 2) were administered to each subject: (A) placebo, (B) chamomile extract capsule, and (C) chamomile tea, in varying order according to randomization assignment. A washout period lasting 7 days between treatments was employed to minimize any carryover effects. Since each subject received all three treatments, there were six possible sequences of treatment (ABC, ACB, BAC, BCA, CAB, CBA). Blocked randomization lists for sequence assignment were computed using SAS© 9.4 Software (SAS Institute Inc., USA). Each participant was randomly assigned one of the six treatment sequences using REDCap software, which provided allocation concealment. The study subjects, research coordinator (co-author D.H.) and the outcome assessor (co-author J.R.) were blinded from placebo versus chamomile extract capsule assignments by way of capsule covering, but they were not blinded from tea consumption.

### Sample size and power calculation

Sample size calculations were performed using PASS Software (NCSS, LLC, USA) with a 3 × 3 cross-over design, Geisser-Greenhouse corrected F-test. The mean prothrombin time derived from a healthy reference sample at our institution was determined to be 11 s with a standard deviation of 0.7 s. We powered the study to detect a 10% change in our study groups, which we deemed as the smallest clinically relevant difference, approximately 1.1 s). Using a range of possible standard deviations, a range of correlations within a subject between 0.4 and 0.5, a power of 80%, and an alpha of 0.017 (adjusted for additional contrasts between treatment groups), the sample size required for the study was 12 participants. Therefore, each treatment sequence appeared twice within the study population.

### Statistical analyses

Demographic and medical history characteristics are described as frequency and percentages for categorical variables. With continuous variables, findings were reported using medians, interquartile ranges, or means and standard deviations (SD) as appropriate. Changes in the endpoints (measurement at completion of 7-day long intervention minus the baseline measurement immediately prior to starting the intervention) across the three study groups were analyzed using linear mixed models. Intervention type (tea, extract capsule, or placebo), randomization sequence, and period were treated as fixed effects, and subjects were treated as random effects. Carryover effects were examined using a period-intervention interaction term. This term was found to be insignificant and therefore removed from the model to reduce over-specification. Covariance structure specification was based on minimizing the Akaike’s Information Criterion. Adjusted contrasts were also examined between each pair of treatments. A *p* value of 0.05 was deemed significant for overall group comparisons, and an adjusted value of 0.017 was considered significant for paired contrasts between the groups. All calculations were performed using SAS© 9.4 Software (Cary, NC, USA).

## Results

**Fig. 3:**
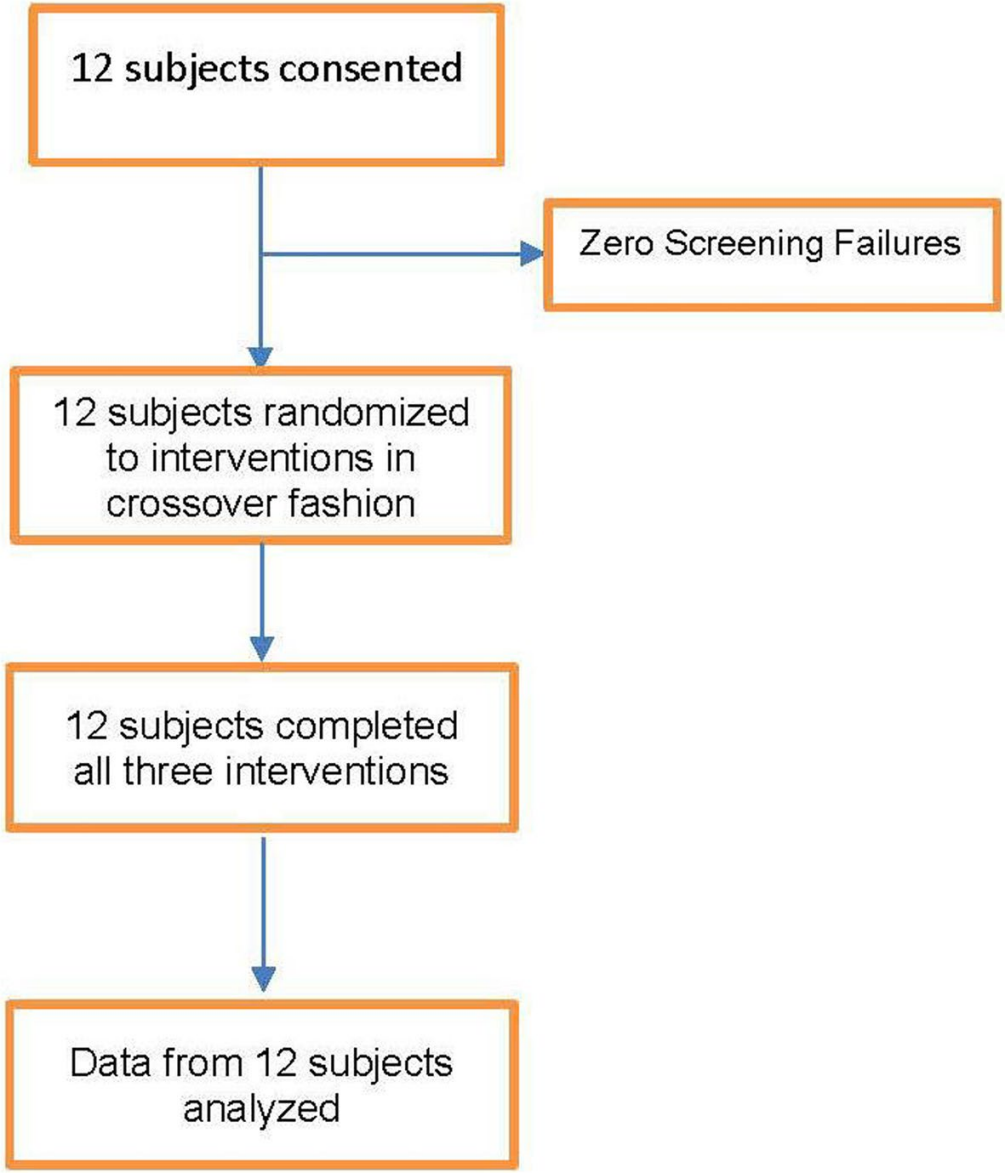
Consolidated standards of reporting trials (CONSORT) flow diagram of research subjects

**Fig. 4:**
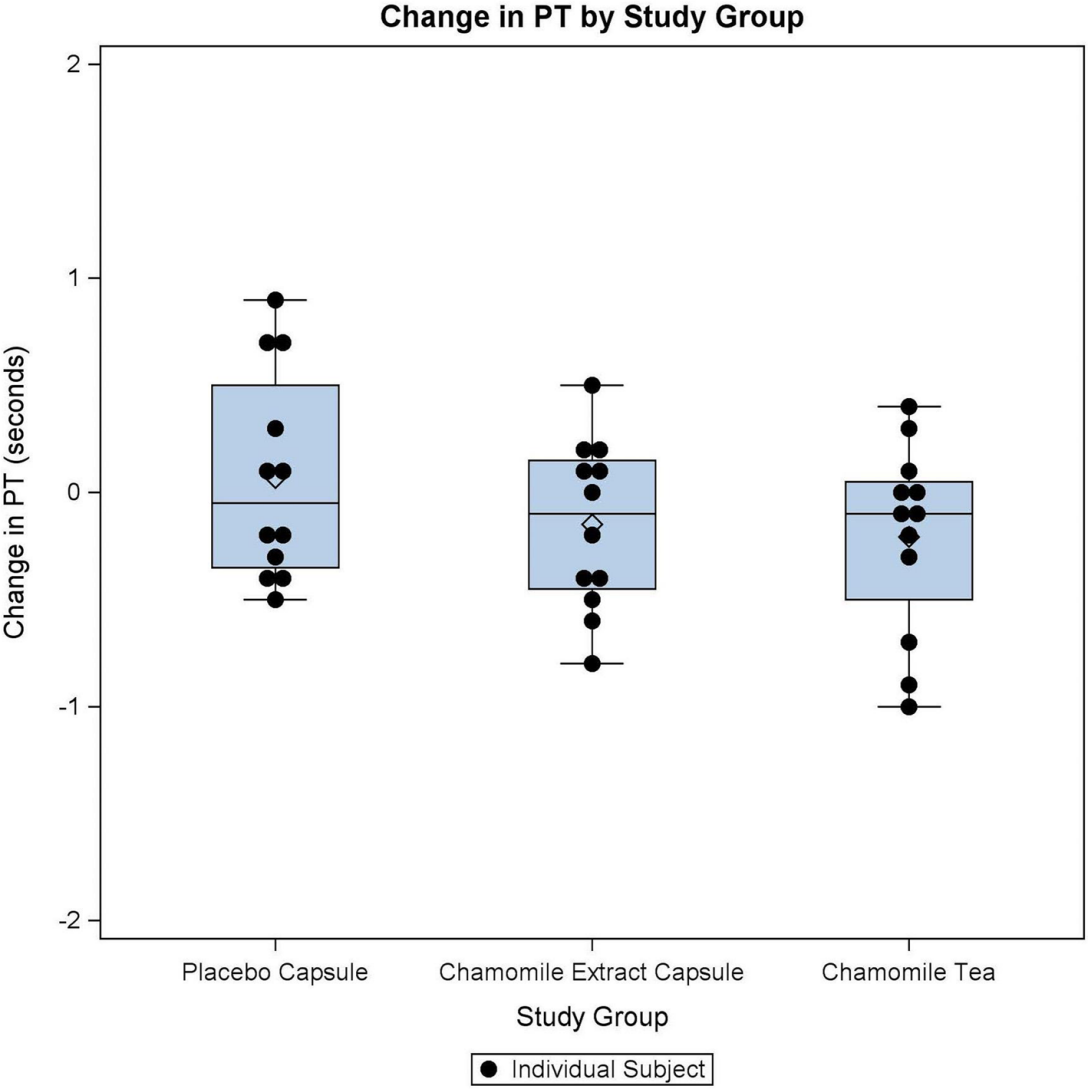
Distribution and average change in prothrombin time by study group. Boxplots showing the change in prothrombin time between the start and end of each intervention. Black dots denote prothrombin time change in each subject within a given study intervention. Blue boxes represent the interquartile range, and the horizontal blue line within the box denotes the median change. Maxima and minima are delimited by the horizontal bars above and below the blue boxes, respectively

A total of 12 subjects were consented and randomized, and all 12 completed each of the three 7-day long interventions (Fig. 3). All data from the 12 subjects were included for analysis. Subjects were mostly female (*n* = 11, 91.7%), with a median age of 30 (Table 1). Adherence to intake of each intervention was high and balanced, with all three arms averaging 20.8 doses (out of a maximum 21 doses) per person. Period, randomization sequence, and carry over effect were not significant in any of the models, indicating a balanced study design with no detectable carry over effect. Change in PT (Fig. 4) was similar in all three groups (*p* = 0.34). On average, participants experienced a small negative change, i.e., decrease, in PT after taking both capsule and tea treatments (Table 2). There was a numerical difference in the change in INR between the three groups (*p* = 0.07). INR was slightly lower after chamomile capsule ingestion compared to the placebo, but it did not reach the pre-set significance of 0.017 for these paired contrasts.

**Table 1:** Subject characteristics

**Demographics**	*n* = 12
Age, years-median (IQR)	30 (25.5, 40)
Gender (female)-*n* (%)	11 (91.7%)
Race	
White	8 (66.7%)
Asian	5 (41.7%)
**Medical history**	
BMI, kg/m^2^-median (IQR)	21.7 (20.5, 24.2)
Medication history	
None	3 (25%)
Hypertension	1 (8.3%)
Hypothyroidism	1 (8.3%)
GERD	1 (8.3%)
Asthma	6 (50%)
**Baseline lab values**	
Hemoglobin, g/dL-median (IQR)	13.1 (12.8, 14.6)
Hematocrit, %-median (IQR)	40.4 (38.8, 44.0)
WBC count, 10^3^/μL-median (IQR)	6.2 (5.5, 7.3)
Platelet count, 10^3^/μL-median (IQR)	292 (253.5, 346)
**Treatment completion (full completion = 21 doses/intervention)**	
Placebo treatment completion	
Full completion-*n* (%)	10 (83.3%)
Average doses per person-mean (SD)	20.8 (0.6)
Capsule treatment completion	
Full completion-*n* (%)	10 (83.3%)
Average doses per person-mean (SD)	20.8 (0.6)
Tea treatment completion	
Full completion-*n* (%)	10 (83.3%)
Average doses per person-mean (SD)	20.8 (0.4)

*IQR* interquartile range, *BMI* body mass index, *GERD* gastroesophageal reflux disease, *WBC* white blood cell, *SD* standard deviation

**Table 2:** Coagulation assay values

	Placebo	Capsule	Tea	Group comparison *P* value	Capsule vs. placebo		Tea vs. placebo		Capsule vs. tea	
					Adjusted difference (95% CI)	*P* value	Adjusted difference (95% CI)	*P* value	Adjusted difference (95% CI)	*P* value
PT (seconds)-mean (SD)										
Baseline^a^	11.1 (0.9)	11.3 (0.7)	11.3 (0.7)	–	–	–	–	–	–	–
Final	11.2 (0.8)	11.1 (0.8)	11.1 (0.8)	–	–	–	–	–	–	–
Change	0.1 (0.5)	− 0.2 (0.4)	− 0.2 (0.4)	0.34	− 0.2 (− 0.6, 0.2)	0.27	− 0.3 (− 0.7, 0.1)	0.17	0.1 (− 0.3, 0.5)	0.77
INR-median (IQR)										
Baseline	1 (0.9, 1.1)	1 (1, 1.1)	1 (1, 1.1)	–	–	–	–	–	–	–
Final	1 (1, 1.1)	1 (1, 1.1)	1 (1, 1.1)	–	–	–	–	–	–	–
Change	0 (0, 0.1)	0 (− 0.1, 0)	0 (0, 0)	0.07	− 0.06 (− 0.1, − 0.01)	0.02	− 0.03 (− 0.07, 0.03)	0.31	− 0.03 (− 0.08, 0.02)	0.18
aPTT (seconds)-mean (SD)										
Baseline	33.6 (2.2)	33.7 (3)	34 (2.4)	–	–	–	–	–	–	–
Final	33.4 (2.3)	33.2 (2.2)	33.4 (2.6)	–	–	–	–	–	–	–
Change	− 0.2 (1)	− 0.5 (1.8)	− 0.6 (1.6)	0.8	− 0.3 (− 1.7, 1.1)	0.8	− 0.5 (− 1.9, 0.9)	0.49	0.2 (− 1.2, 1.5)	0.8
TT (seconds)-mean (SD)										
Baseline	20.9 (1.5)	22.1 (1.6)	21.1 (1.5)	–	–	–	–	–	–	–
Final	22.3 (1.4)	22 (1.1)	21.9 (1.2)	–	–	–	–	–	–	–
Change	1.4 (1.4)	− 0.1 (1.8)	0.8 (1.6)	0.08	− 1.5 (− 2.9, − 0.2)	0.03	− 0.6 (− 1.9, 0.7)	0.35	− 0.9 (− 2.2, 0.4)	0.16
RT (seconds)-mean (SD)										
Baseline	15.1 (0.8)	15.3 (0.9)	15.1 (1.2)	–	–	–	–	–	–	–
Final	14.9 (1)	15.2 (0.9)	15.1 (0.7)	–	–	–	–	–	–	–
Change	-0.1 (0.9)	0 (1.1)	0.1 (0.9)	0.8	0.1 (− 0.6, 0.9)	0.7	0.2 (− 0.5, 0.9)	0.56	− 0.1 (− 0.8, 0.6)	0.79
FG (mg/dL)-mean (SD)										
Baseline	418.3 (69.8)	379.8 (53.2)	411 (72.3)	–	–	–	–	–	–	–
Final	400.1 (47.3)	393.2 (54)	396.4 (70.2)	–	–	–	–	–	–	–
Change	− 18.3 (63.7)	13.4 (43.4)	− 14.6 (42.6)	0.2	31.7 (− 8.7, 72.1)	0.1	3.7 (− 36.7, 44.1)	0.85	28.0 (− 12.4, 68.4)	0.16

*SD* standard deviation, *IQR* interquartile range, *PT* prothrombin time, *INR* international normalized ratio, *aPTT* activated partial thromboplastin time, *TT* thrombin time, *RT* reptilase time, *FG* fibrinogen level

^a^The baseline value (just prior to starting an intervention), final value (just after completion of a particular intervention), and the change (final minus initial values) are shown

This study was not rigorously powered to assess clinically significant bleeding. Nevertheless, very few changes occurred in bruising or bleeding from beginning to end of any of the treatments (Table 3).

**Table 3:** Bleeding questionnaire

	Placebo	Capsule	Tea
Bruise easily *n* (%)			
Baseline	0 (0%)	0 (0%)	0 (0%)
Post-intervention	0 (0%)	0 (0%)	1 (8.3%)
Gum bleeding-*n* (%)			
Baseline	0 (0%)	1 (8.3%)	0 (0%)
Post-intervention	0 (0%)	0 (0%)	0 (0%)
Other bleeding-*n* (%)			
Baseline	1 (8.3%)	0 (0%)	0 (0%)
Post-intervention	0 (0%)	0 (0%)	0 (0%)

Questionnaire responses: Subjects completed a questionnaire before and after each of the three 7-day study interventions. Any self-reported occurrence was counted, permitting the possibility that a single subject could report multiple events across serial questionnaire assessments. The number of self-reported occurrences for each questionnaire item (bruising easily, onset of gum bleeding, or other bleeding) is shown. The percentage of self-reported occurrences relative to the number of study subjects (12) is provided in parentheses

## Discussion

Concerns that chamomile might cause increased bleeding risk during surgery have led to recommendations against the preoperative intake of chamomile, even in the vast majority of individuals who do not take anticoagulants, e.g. warfarin (Mount Sinai et al. 2022; Wong 2022; Abebe 2019). A case report largely serves as the basis for this concern. Since that case report, controlled laboratory evidence for a direct anticoagulant effect by chamomile has been lacking. Our randomized, placebo-controlled crossover study found no adverse effect by chamomile (either tea or powder extract administered 3 times per day for 7 days) on prothrombin time (primary endpoint) or any of the secondary endpoints.

Recommendations to withhold chamomile prior to surgery appear to rest primarily on a single case report. This case report described a 70-year-old woman taking warfarin who presented to the hospital with multiple spontaneous internal hemorrhages and INR up to 7.9 after consuming 4–5 cups of chamomile tea daily. After excluding medication errors or platelet defects as causes, the authors speculated that the patient’s coagulopathy may have resulted from a synergy between coumarin-like compounds in chamomile and warfarin (Segal and Pilote 2006). Herb-drug interactions were deemed less likely to explain the supratherapeutic INR as chamomile primarily inhibits the cytochrome P450 (CYP) isozyme, CYP1A2, that metabolizes the weaker R-enantiomer of warfarin (Ganzera et al. 2006). Furthermore, chamomile constituents only weakly inhibit the CYP2C9 isozyme that metabolizes the highly active S-enantiomer of warfarin (Kaminsky and Zhang 1997). Interestingly, the authors did not discuss the potential role of the patient’s respiratory tract infection and concurrent use of alendronate, amiodarone, and camphor lotion (which may contain salicylates) on warfarin sensitivity (Clark et al. 2014; Qian et al. 2020; McDonald et al. 2012; Joss and LeBlond 2000; Yarnell and Abascal 2009).

The findings from our study show no change in PT or other measured coagulation assays despite 7 days of chamomile intake, which suggests that the supratherapeutic INR encountered in the case report is more likely due to a process different from synergy between coumarin-like compounds and warfarin.

Coumarins describe a large group of naturally occurring benzopyrone phytochemicals with a wide range of biologic activities (Matos et al. 2015; Venugopala et al. 2013). The most well-known pharmacologic derivative from the coumarins is the anticoagulant warfarin (Triplett 1998). Chamomile is known to contain several coumarin compounds that could theoretically possess anticoagulant properties (Tschiggerl and Bucar 2012; Petruľová-Poracká et al. 2013; Ramesh and Pugalendi 2007). However, coumarins as a broad class of compounds often do not produce anticoagulant activity (Yarnell and Abascal 2009).

Prothrombin time has been utilized as a sensitive assay for anticoagulant activity by vitamin K antagonists such as warfarin (Triplett 1998; Roshal and Reyes Gil 2019; Kamal et al. 2007). The results from our study indicate that chamomile, when consumed as a chamomile extract or tea, does not have a large independent effect on a coagulation process evaluated by the PT assay. Additionally, the lack of change in the aPTT observed in our study suggests against a heparin-like effect (Roshal et al. 2019a). The absence of changes in TT and RT indicate that chamomile does not inhibit thrombin or induce acquired dysfibrinogenemias (Roshal et al. 2019b; Francischetti et al. 2019; Cunningham et al. 2002). Since FG levels may influence PT, aPTT, TT, and RT, our study assessed changes in FG levels as a possible confounder (Cott et al. 2002). However, we found no changes in FG levels after chamomile intake.

Our study did not examine antiplatelet effects associated with tea or extract capsule intake. One in-vitro study investigated the potential antiplatelet effects of polysaccharide-polyphenolic conjugates extracted from chamomile (Bijak et al. 2013) However, the chamomile dosages utilized to generate a plasma concentration of 100 μg/mL with these polyphenolic conjugates were very high. Assuming 100% bioavailability, a subject would need to consume a minimum of 7 g chamomile flowers whole (equivalent to 7 tea bags) in a single dose to suffer a potential antiplatelet effect, a notably higher dose than each tea serving used in our study (Manach et al. 2004).

Our study has several limitations. It is a single center study, which limits its external validity. Our study *excluded* subjects actively taking warfarin, thus it is theoretically possible that chamomile might still potentiate warfarin effects. Additionally, our final study population consisted primarily of young females, which might limit the generalizability of our study results. Since our study examined 7 days of chamomile intake, we cannot rule out an anticoagulant effect that appears only after longer durations of chamomile ingestion. A protracted onset time, however, would be less likely if the coumarin-like compounds in chamomile exhibited a similar vitamin-K antagonist effect like warfarin since warfarin typically requires only days to demonstrate prothrombin prolongation.

Another limitation includes the possibility that the study design may fail to detect true PT prolongation after chamomile exposure if the half-life of anticoagulation effect is short. Also, since PT alone does not unequivocally predict perioperative bleeding risk in isolation, our study results cannot completely exclude an increased clinical bleeding risk after chamomile ingestion.

Our study has several strengths. The randomized, placebo-controlled crossover design is an important strength of our study. In the cross-over design subjects served as their own controls, which significantly increases the study’s power. Another strength of the study is our use of two different chamomile formulations (liquid tea and extract capsule). It is reassuring that the results were similar for both formulations tested. We also chose a widely used, commercially available chamomile tea to increase the study’s generalizability. Another strength is partial blinding from chamomile extract versus placebo capsules, though bias is unlikely given the objective endpoints of standardized coagulation tests. Also of note, we used high doses for both our chamomile formulations to safely ensure sufficient exposure to active ingredients. For example, as described in our methods, each cup of chamomile tea (ingested three times per day) was made from three tea bags, making every serving quite strong. Patients are unlikely to consume a stronger tea than this study’s preparation. Furthermore, adherence to the study protocol, namely capsule intake, was high. Use of multiple additional screening assays beyond the PT improved the experimental sensitivity toward identifying a chamomile-related anticoagulation effect.

In conclusion, this randomized, placebo-controlled, crossover trial of healthy subjects found no deleterious effect on the prothrombin time or any of the prespecified secondary endpoints of anticoagulation after consuming chamomile as a tea or extract capsule. These findings challenge the notion that chamomile should be held routinely in the perioperative period by patients who are not taking warfarin or other anticoagulants. In patients who are taking warfarin or other anticoagulants in the preoperative period, more research is needed as our study did not focus on this smaller group of individuals.

## Data Availability

The datasets used and/or analyzed during the current study are available from the corresponding author on reasonable request.

## Abbreviations

aPTT: Activated partial thromboplastin time
BMI: Body mass index
CONSORT: Consolidated Standards of Reporting Trials
CYP: Cytochrome P450 Enzyme
FG: Fibrinogen
GERD: Gastroesophageal reflux disease
INR: International normalized ratio
IQR: Interquartile range
IRB: Institutional Review Board
PT: Prothrombin time
SD: Standard deviation
TT: Thrombin time
WBC: White blood cell count
